# Identification and quantification of the molecular species of bilirubin BDG, BMG and UCB by LC‒MS/MS in hyperbilirubinemic human serum

**DOI:** 10.1371/journal.pone.0313044

**Published:** 2024-11-19

**Authors:** Stephany M. Castillo-Castañeda, Liliana Rivera-Espinosa, Josefina Gómez-Garduño, Jacqueline Cordova-Gallardo, Juan Luis Chávez-Pacheco, Nahum Méndez-Sánchez

**Affiliations:** 1 Liver Research Unit, Medica Sur Clinic & Foundation, Mexico City, Mexico; 2 Medical, Dental and Health Sciences Master and Doctorate Program, National Autonomous University of Mexico, Mexico City, Mexico; 3 Pharmacology Department, National Pediatric Institute, Mexico City, Mexico; 4 Faculty of Medicine, National Autonomous University of Mexico, Mexico City, Mexico; 5 Hepatology, General Surgery Department, General Hospital Dr. Manuel Gea González, Mexico City, Mexico; University of Michigan, EGYPT

## Abstract

**Background and aims:**

Unconjugated bilirubin (UCB) is a byproduct of the heme group that indicates irregularities in the metabolism of several important biological molecules, such as hemoglobin. UCB is processed by hepatic UGT1A1, which catalyzes its conjugation to the metabolites bilirubin diglucuronide (BDG) and bilirubin monoglucuronide (BMG). The serum concentrations of BDG and BMG may indicate liver injury or dysfunction. The aim of this study was to standardize and validate a method for the identification and simultaneous quantification of BMG, BDG and UCB by LC‒MS/MS.

**Methods:**

Liquid‒liquid extraction allows the separation of UCB, BMG and BDG from the serum of healthy subjects or patients with liver injury. Detection and quantification were performed using an LC‒MS/MS method. Compound separation was achieved with a BEH-C18 column at 40°C. The mobile phase was prepared with 5 mM ammonium acetate (pH 6) and acetonitrile, and a flow gradient was applied.

**Results:**

This is the first study to directly quantify BMG and UCB levels in human serum; no postcalculations or correction factors are needed. However, BDG quantification requires calculations and a correction factor. We identified the molecular species with ionic transitions m/z^1+^ 585.4 > 299.2 for UCB, 761.3 > 475.3 for BMG, 937.3 > 299.5 for BDG and mesobilirubin 589.4 > 301.3 (IS).

**Conclusion:**

The procedures used in this study allowed the simultaneous identification and quantification of the molecular species of bilirubin, BDG, BMG and UCB. Analysis of the serum levels in patients with hyperbilirubinemia revealed that patients with acute-on-chronic liver failure had elevated levels of these species.

## Introduction

Bilirubin is the breakdown product of the heme group, a tetrapyrrole ring with an iron atom in the center (iron protoporphyrin IX). This group is present in hemoglobin and other hemoproteins, including myoglobin, cytochrome P-450, catalase, peroxidase, and mitochondrial cytochromes. Bilirubin is formed by cleavage of the porphyrin ring. First, hemoglobin is cleaved into free globin chains, and the heme group is engulfed by macrophages, mainly in the spleen. The heme is then oxidized and reduced by two enzymes, microsomal heme oxygenase and cytosolic biliverdin reductase A, to form unconjugated bilirubin (UCB). Once UCB reaches the hepatocyte cytosol, it becomes a substrate for the enzyme uridine diphosphate glucuronosyltransferase (UGT1A1), which catalyzes the esterification of bilirubin with glucuronic acid to synthesize bilirubin diglucuronide (BDG) and a small amount of bilirubin monoglucuronide (BMG). Synthesis of glucuronides is essential for efficient biliary excretion of bilirubin and further reduces its toxicity [1–3]. After bilirubin is excreted into the canaliculi and ultimately the intestinal tract, it is further metabolized by intestinal bacteria to form a compound called urobilinogen, which can be reabsorbed in the intestine and excreted in the urine. Eventually, intestinal urobilinogen is converted to stool pigments such as stercobilin [1, 4].

The liver plays a central role in bilirubin metabolism and, under normal conditions, processes bilirubin efficiently without causing significant changes in the serum bilirubin level. However, when the liver suffers severe damage, such as necrotic or apoptotic cell death, which disrupts metabolism by altering conjugation and impairing uptake or excretion, it loses its ability to properly regulate bilirubin, resulting in elevated serum bilirubin levels, known as hyperbilirubinemia [5, 6]. Two primary types of hyperbilirubinemia are distinguished: unconjugated and conjugated, and both types result in jaundice when the serum bilirubin concentration exceeds 3 mg/dL. Unconjugated hyperbilirubinemia is not strongly linked to liver disease and occurs when there is excess bilirubin before it reaches the liver for conjugation. This is commonly due to increased red blood cell destruction or genetic conditions such as Gilbert syndrome. In contrast, conjugated hyperbilirubinemia arises when the liver is unable to excrete bilirubin properly. This is typically observed in patients with liver disorders and varies on the basis of the progression and duration of the disease [4, 6, 7]. However, contrary to the traditional view that bilirubin is a purely toxic substance that impairs liver function, recent evidence highlights its multifaceted role. Bilirubin is not just a waste product but also a complex molecule with regulatory, antioxidant, anti-inflammatory and cytoprotective properties. These diverse functions have been extensively documented, challenging the outdated notion that bilirubin is solely deleterious and underscoring its broader importance in both liver health and disease [8–10].

In clinical practice, the measurement of circulating bilirubin is critical because of the multiple factors involved in its metabolism, including enzymatic reactions in heme catabolism, membrane transport systems for hepatic excretion, and the absorption of bilirubin glucuronide from the intestine [4, 6]. Given the complexity of these processes, bilirubin serves as an important marker of liver disease, reflecting progressive cellular dysfunction. Its measurement is critical in guiding clinical decisions, providing valuable insight into the metabolic capacity and overall health of the liver [4, 11–21]. Analysis of bilirubin (conjugated and unconjugated) can be difficult because of the lack of standard bilirubin glucuronides, so methods such as in vitro glucuronidation (the conjugation of bilirubin with glucuronic acid) have been used to characterize BMG and BDG [22, 23]. Conventional methods for bilirubin measurement, including the widely used diazo reaction, indirectly quantify unconjugated bilirubin by subtracting conjugated bilirubin (BMG and BDG) from total bilirubin. Other methods, such as enzymatic, chemical, spectrophotometric, oxidation, or high-performance liquid chromatography (HPLC), have also been used. While these methods provide a general estimate of bilirubin levels, they lack precision and specificity, particularly in distinguishing between different bilirubin glucuronides. As a result, they may overlook key molecular differences that are critical for understanding the underlying pathology of liver diseases [22, 24–30].

In this context, liquid chromatography coupled with tandem mass spectrometry (LC‒MS/MS) has emerged as a powerful tool for the quantification and characterization of bilirubin metabolites. This method allows accurate identification of bilirubin and its glucuronides with high sensitivity and specificity, offering a detection limit as low as 1.8 nM. LC‒MS/MS is particularly useful for in vitro metabolism studies evaluating enzyme systems such as human liver microsomes (HLMs), rat liver microsomes (RLMs) and recombinant enzymes (rUGT1A1), allowing the analysis of bilirubin glucuronidation kinetics under controlled conditions, facilitating the understanding of differences between bilirubin molecular species and the evaluation of potential drug‒drug interactions that could inhibit UGT1A1 activity [24, 31]. The ability of LC‒MS/MS to quantify both BMG and BDG makes it a reliable and sensitive technique.

The aim of this study was to develop a sensitive and specific method for the identification and quantification of molecular species of bilirubin, BMG, BDG and UCB using LC‒MS/MS through in vitro glucuronidation reaction, and by glucuronides extracted from the serum of patients with hyperbilirubinemia. These biomarkers may serve as indicators of the degree of liver damage.

## Methods

### Reagents and standards

Pure standards of bilirubin and mesobilirubin as internal standards (I.S.) were purchased from Santa Cruz (Santa Cruz, Inc., CA, USA). Standards of propofol, dexmedetomidine, ceftriaxone and prednisone were all purchased from MP Biomedicals (Fountain Pkwy, Solon OH, USA). LC‒MS grade acetonitrile (ACN) and methanol (MeOH) were obtained from JT Baker. Ammonium acetate, Tris-HCl buffer, KCl, NaOH, CuSO_4_, MgCl_2_, dimethyl sulfoxide (DMSO), EDTA and phosphate-buffered saline (PBS) were obtained from Merck (Darmstadt, Germany®), and ascorbic acid, phenylmethylsulfonyl fluoride (PMSF), Na_2_CO_3_, Na^+^ and K^+^ tartrate, Folin-Ciocalteu, dithiothreitol (DTT) and protease inhibitor (cOmplete®) were obtained from Sigma Aldrich® (St. Louis MO, USA). Glycerol was obtained from Invitrogen (Thermo Fisher Scientific, MA, USA). Alamethicin, D-saccharolactone, and uridine-5’-diphosphoglucuronic acid (UDPGA) were purchased from Santa Cruz (Santa Cruz, Inc., CA, USA). For all the solutions and dilutions, bidistilled water filtered through a Milli-Q system (Millipore, Molsheim, France®) was used.

### Chromatographic and spectrometric conditions

Ultrahigh-performance liquid chromatography (UPLC) coupled with tandem mass spectrometry (Quattro Micro^TM^; Waters Micromass, Manchester, UK) was used. The spectrometer was operated in electrospray positive ionization mode (ESI+) and multiple reaction mode monitoring, and the ionic transitions were m/z^1+^ 585.4 > 299.2 for UCB, mesobilirubin 589.4 > 301.3, 761.3 > 475.3 for BMG and 937.3 > 299.5 for BDG. The cone (V)/collision energy (eV) conditions were 35/25 for bilirubin, 30/30 for mesobilirubin, 40/20 for BMG and 40/40 for BDG, with a residence time of 0.1 s for all. The desolvation gas flow was set at 650 L/h at 350°C, while the cone gas flow was set at 50 L/h. The source temperature was maintained at 120°C. The data obtained were processed with MassLynx1 4.1 software.

Compound separation was performed through a BEH-C18 column (2.1 × 50 mm, 1.7 μm) maintained at 40°C. The autosampler was set at 21°C, and the mobile phase consisted of 5 mM ammonium acetate (pH 6.0; solvent A) and ACN (solvent B) with a linear flow gradient (Table 1). The running time was 7 minutes.

**Table 1:** Mobile phase flow gradient.

Time (min)	Flow (mL/min)	A (%)	B (%)
Initial	0.125	30	70
1.0	0.150	30	70
1.6	0.150	20	80
2.0	0.150	40	60
2.4	0.150	50	50
2.6	0.150	40	60
2.8	0.150	70	30
3.0	0.150	80	20
3.3	0.150	80	20
3.8	0.125	70	30
5.0	0.150	50	50
6.0	0.150	30	70
7.0	0.125	30	70

Compound separation was performed with a linear flow gradient. Solvent A, 5 mM ammonium acetate, pH 6.0; solvent B, acetonitrile.

### Standards and controls

Stocks of 30 mM UCB and 40 mM solutions of mesobilirubin (I.S.) were prepared daily in DMSO and stored in amber vials. Work solutions for UCB were prepared (20X) in DMSO, protected from light, to obtain solutions for calibrators at 200, 400, 600, 800, 1000 and 1200 μM concentrations. Low (LQC), medium (MQC), and high (HQC) quality control UCB solutions were prepared at 300, 700 and 900 μM, respectively.

Independent calibration curves were prepared with serum from volunteers exposed for 6 hours to fluorescent light (white light) to deplete total bilirubin. The UCB calibration curve consisted of 10, 20, 30, 40, 50 and 60 μM for calibrators and 15, 35 and 45 μM for quality controls, which were prepared by adding 950 μL of serum and 50 μL of working solution. The curves were prepared fresh daily. On the other hand, the preparation of glucuronide calibrators (BMG and BDG) was not possible with commercially obtained standards. BDG is sold commercially, but the import process was difficult for us, so calibrators for this analyte could not be made. In the case of BMG, the standard BMG is not commercially available, so it was obtained through three techniques.

### Production of bilirubin conjugates

Three different methods were tested to obtain bilirubin conjugates, two in vitro assays: the first uses microsomes from human liver cells (HLMs), and the second uses microsomes from rat liver (RLMs). Preparation of HLMs and RLMs, and the specific biochemical reactions for BMG and BDG in vitro synthesis are described in the following sections. In the third method, BMG and BDG were obtained from serum samples from patients with hyperbilirubinemia (in vivo assay), providing a real-life context for comparison.

#### Microsomes from hepatocyte culture (HLMs)

The HepG2 hepatocyte cell line was maintained in Dulbecco’s modified Eagle’s medium containing Nutrient Mix F-12 (DMEM-F12) supplemented with 10% fetal bovine serum and 1% antibiotics (penicillin/streptomycin). The cells were incubated in 75 cm^2^ flasks in humidified air containing 5% CO_2_ at 37°C for 3 days, DMEM-F12 medium was replaced daily. After the cells reached 90% confluence, they were passaged by treatment with 0.25% EDTA-treated trypsin and centrifugation at 2500 rpm for 5 minutes. The supernatant was discarded, and the cell pellet was removed. The cells were stained with trypan blue and counted in a Neubauer chamber. HepG2 cells were exposed to different omeprazole concentrations (10, 25, 50, 100, 200 and 500 μM) to induce synthesis of the UGT1A1 enzyme [32]. Viability assay, with crystal violet staining technique, was performed after 24 h of omeprazole exposure. A negative and a positive control (oxidative stress with 300 mM ascorbic acid and 30 mM copper sulfate) were also included. Viability was assessed by reading the absorbance of the plates stained with crystal violet in a spectrophotometer at 590 nm and comparing the results to those of the positive and negative controls. Maximum induction of UGT1A1 was achieved with 50 μM omeprazole, therefore, the next cultures of HepG2 cells were performed in DMEM-F12 medium supplemented with 50 μM omeprazole.

After incubation, approximately 3x10^5^ cells were washed twice with cold PBS. Cold lysis buffer (100 mM Tris-HCl, 1.15% KCl, 1 mM EDTA, 0.1 mM PMSF, and 0.1 mM DTT) was then added, and the cells were detached with a rubber scraper. The cells were transferred to sterile 15 mL conical tubes and centrifuged at 2500 rpm for 5 minutes. The cell pellet was resuspended in storage buffer containing 100 mM Tris-HCl, 1 mM EDTA, 0.1 mM EDTA-PMSF, 0.1 mM DTT with 20% glycerol and cOmplete protease inhibitor, lysed in a glass homogenizer and then centrifuged at 9,000 rpm for 20 minutes at 4°C. The supernatant was homogenized again, centrifuged at 41,300 rpm for 60 minutes at 4°C in an XL-90 ultracentrifuge (Beckman). The pellet was subsequently resuspended in storage buffer [33].

#### Microsomes of rat liver (RLMs)

Male Wistar rats were sacrificed, and their livers were removed and placed in cold lysis buffer on ice. The livers were obtained via donation from animals in the control group of protocol INP 048/2022, which was approved by the Research, Biosafety and Animal Care Committees of the National Institute of Pediatrics. The animals were euthanized by decapitation. Anesthesia was induced with sodium pentobarbital at 200 mg/kg. The livers were minced with scissors, placed in a tube, and homogenized with a Bio-Gen PRO 200 homogenizer. The homogenate was centrifuged at 9,000 rpm for 20 minutes at 4°C. The supernatant was homogenized again and centrifuged at 41,300 rpm for 60 minutes at 4°C. The pellet was washed three times with storage buffer and then transferred to Eppendorf tubes containing storage buffer supplemented with cOmplete protease inhibitor. Protein quantification of HLMs and RLMs was performed according to the Lowry method [34].

#### Monoglucuronide and diglucuronide bilirubin conjugation

Bilirubin was incubated with HLMs or RLMs, which had been treated with alamethicin (50 μg/mg protein) for 15 minutes on ice. Then, MgCl_2_ (10 mM) and D-saccharolactone (5 mM) were added to Tris-HCl buffer (0.1 M, pH 7.4); finally, UDPGA (3 mM) was added. Triplicate reactions were performed in amber Eppendorf tubes with a final volume of 200 μL. The microtubes were incubated for 10, 15, 20, 30 or 45 minutes at 37°C in the dark in a TR100-G thermoshaker. The samples were prepared as described in the “Sample processing” section.

Enzyme kinetic data for bilirubin glucuronidation were analyzed via the Michaelis‒Menten model in Graph Pad Prism (v10.2.0, GraphPad, Inc.). Enzyme kinetic parameters were determined for each incubation, and the results are expressed as the mean ± standard deviation (SD). Additionally, the ratio of Vmax/km was calculated as the intrinsic clearance (CLint) in the incubations.

### Bilirubin conjugates obtained from patient samples

The serum of six patients was pooled, and 200 μL was processed to extract bilirubin and its conjugates as described in the “Sample processing” section. The chromatographic conditions previously described were used on an Acquity UPLC with a UV/Vis detector. BMG was detected at 450 nm for collection, as this is a nondestructive method unlike mass spectrometry and allows BMG recovery. The sample output was collected during the first 4.5 min, following the retention time for mass spectrometry. It was collected directly from the UPLC instrument in amber Eppendorf tubes, maintained on ice, and subsequently frozen at -80°C. A total of 500 μL of sample was collected for subsequent evaporation of the mobile phase with N_2_. The Turbo Vap LV was used under the following conditions: nitrogen was applied at a pressure of 4 bar with a flow rate of 0.7 L/min and a temperature of 30°C for two hours. Next, the dry powder was resuspended in 184 μL of MeOH and DMSO (75:25 v/v), and 16 μL of ascorbic acid (100 mM) was added. To determine the concentration, a 1:50 dilution was transferred to a cuvette (1 cm path light) and read in a Multiskan Go spectrometer at 450 nm. The concentration was calculated with the transformed Beer‒Lambert law formula: c = εb/A, where **ε** is the molar absorptivity of the molecule, **b** is the path length, and **c** is the molecule concentration.

In addition, the LC‒MS/MS bilirubin curve was used to determine the concentration of BMG. Two ranges of calibration curves for BMG were generated to characterize the concentrations of both healthy individuals and patients, the first, named High Range with concentrations of 3.84, 7.62, 15.25, 30.50, 61.01, and 122.02 μM and LQC, MQC and HQC at 5.71, 22.87, and 91.51 μM, respectively. The second, named Low Range, was prepared using concentrations of 0.89, 1.78, 3.57, 7.15, 14.30, and 28.60 μM, with LQC, MQC and HQC at 1.39, 5.58, and 21.50 μM, respectively. All the curves were prepared fresh on the same day.

### Sample processing

Patient samples, calibrators, and quality controls were prepared by pipetting 200 μL of serum into a 1.5 mL microtube. Then, 20 μL of I.S. (40 μM), 550 μL of a mixture of MeOH and DMSO (80:20 v/v), and 50 μL of ascorbic acid (100 mM) were mixed vigorously by vortexing for 20 seconds and then in an ultrasonic bath for 5 minutes. The sample was subsequently centrifuged for five minutes at 10,000 rpm. The supernatant was removed and placed in UPLC vials, and 10 μL was injected into the chromatographic system.

### Method validation

Validation was performed by the following parameters: selectivity, matrix effect, carryover, linearity, accuracy, precision and stability, as described in the Mexican Official Standard NOM177-SSA1-2013 [35], which is in accordance with international guidelines for bioanalytical methods [36, 37].

#### Selectivity and carryover

Selectivity was evaluated by comparing the chromatograms of the drugs administered concomitantly with the matrix blank and the LQC signals of UCB and BMG. The carry-over was conducted by injecting the LQC, HQC, and three blanks in succession.

#### Matrix effect

This parameter was determined by extracting blank samples in serum, and at the end of the process, solutions of the analyte and I.S., quality controls (LQC, MQC and HQC) were added, and their responses were compared with those of each of them separately. For each unit, a normalized matrix factor was obtained according to the following formula:

The normalized matrix factor (NMF) was calculated via the following formula:

$NMF=\frac{\left(Response\;of\;the\;analyte\;in\;the\;matriz\;/Response\;of\;the\;internal\;standard\;in\;the\;matrix\right)}{\left(Response\;of\;the\;analyte\;in\;solution/Response\;of\;the\;internal\;standard\;in\;solution\right)}$

The acceptance criterion for the matrix effect was a coefficient of variation of less than 15% for NMF.

#### Linearity

Six concentrations of UCB and BMG were analyzed for at least 3 continuous days to establish the relationship between concentration and response.

#### Accuracy and precision

Intraday tests were performed on five series of quality controls: the lower limit of quantification (LLQ), LQC, MQC and HQC. All quality controls were prepared daily. For interday assays, quality controls were injected in triplicate on three consecutive days.

#### Stability

Stability was evaluated by subjecting the sample to different storage conditions.

### Method application

The method was applied to serum samples collected from patients recruited between April 2023 and December 2023 at the General Hospital ’Dr. Manuel Gea González’ and Hospital Medica Sur. Both hospitals obtained approval for sample collection from their respective Research and Ethics Committees, with registration numbers CEI-168-2023 and 11-2021-CEI-111. Written informed consent was obtained from all patients and healthy volunteers. For each participant, 3 ml of whole blood was drawn for serum extraction. Four patient groups were established: those with acute‒on-chronic liver failure (ACLF; n = 10), hepatic encephalopathy (HE; n = 10), compensated cirrhosis (LC; n = 10), and a control group of healthy individuals (n = 10).

### Statistical analysis

The validation parameters were evaluated using mean data, standard deviations, and coefficients of variation. The Wilcoxon signed-rank test was used to compare the concentrations obtained with the reported correction factor and the concentrations obtained after interpolation of our curves prepared with the corresponding calibrators. Since we compared the two methods, we used the Benjamini‒Hochberg (BH) procedure for p values. This provides more flexibility and a greater chance of detecting true positives while still controlling the false discovery rate.

## Results

### Synthesis of conjugates by in vitro or in vivo assays

**Fig 1:**
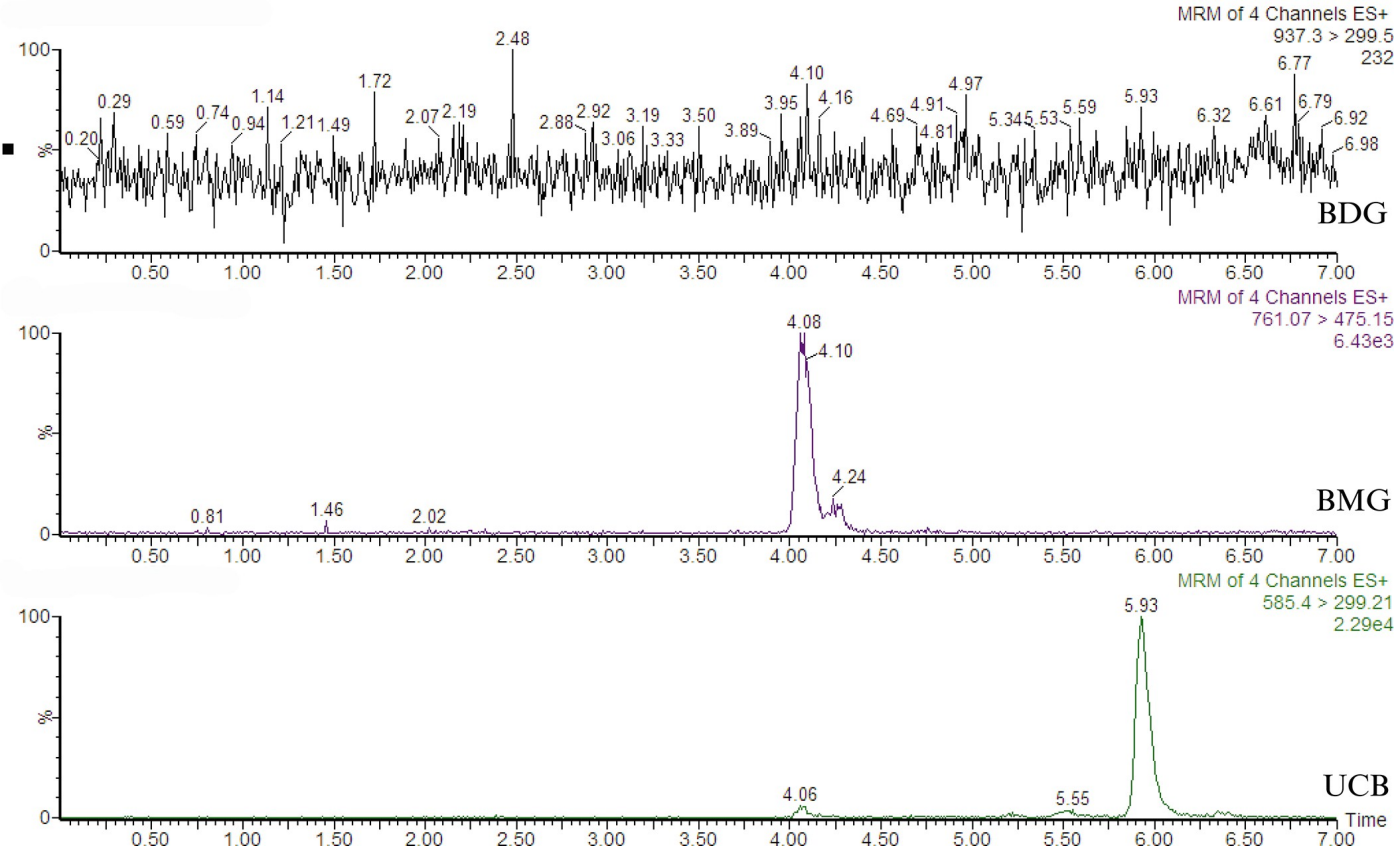
Representative chromatogram showing the peaks resulting from the incubation of rat liver microsomes. The cells were incubated with unconjugated bilirubin (UCB) for 20 minutes to obtain bilirubin monoglucuronide (BMG) and bilirubin diglucuronide (BDG). The transitions for each molecular species of bilirubin were as follows: BDG m/z 937.33 > 299.5, BMG 761.30 > 474.30, and UCB 585.27 > 299.21. The retention times were as follows: UCB, 5.93 min; BMG, 4.08 min; and BDG, not observed.

**Fig 2:**
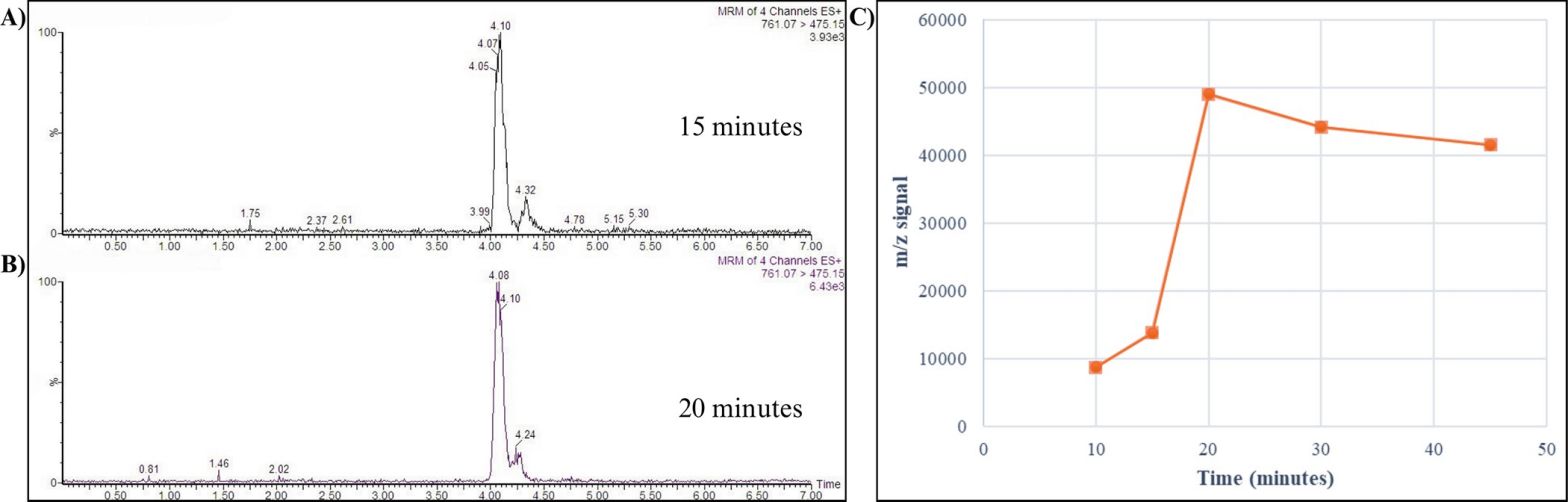
Incubation for the bilirubin glucuronidation reaction. Panel A, 15 min; Panel B, 20 min. The production profile of bilirubin monoglucuronide, following the m/z signal, is depicted in Panel C.

The synthesis of BMG, verified by mass spectrometry, shown that this conjugate was produced in both in vitro assays, but BDG was not successfully obtained. When the results of the two in vitro assays were compared, BMG was optimally synthesized, but BDG was not (Fig 1). Additionally, the comparison of the production of BMG in both assays demonstrated that the RLM system was better than the HLM system. The best glucuronidation conditions were obtained with 75 μM bilirubin and incubation for 20 minutes (Fig 2).

**Fig 3:**
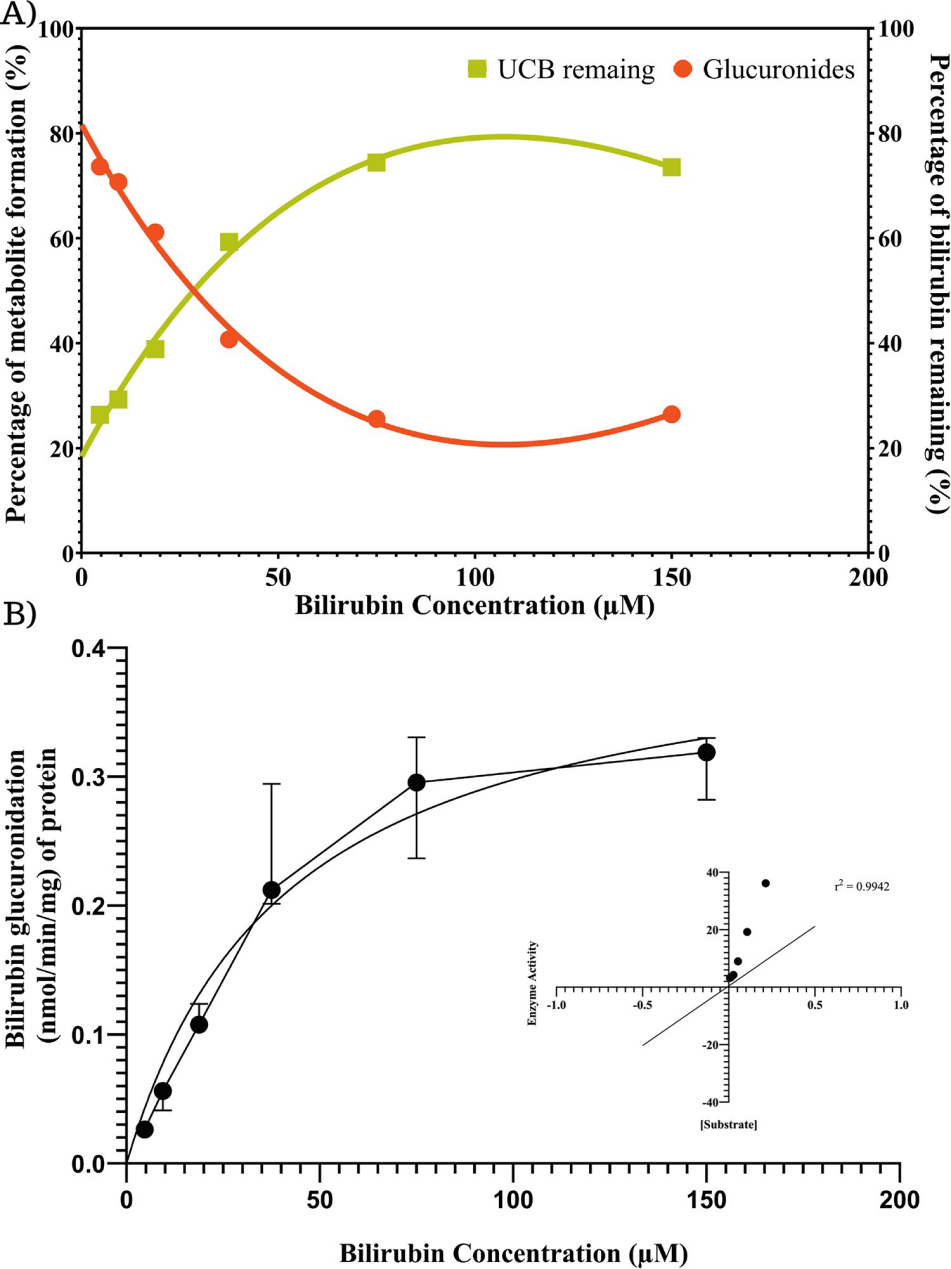
A) Plot of substrate concentration (UCB) vs. glucuronidation (synthesis of metabolites) and remaining UCB at the end of the reaction. B) Kinetic profiles of bilirubin glucuronidation using rat liver microsomes. The microsomal or UGT1A1 protein concentration was 6 μg/mL, and the samples were incubated for 20 min. Each data point represents the mean of three replicates. Kinetic parameters were calculated by the Lineweaver‒Burk plot (r^2^ value of 0.9942).

The synthesis of BMG was assayed with different concentrations of UCB, and the results showed that BMG formation was greater at low substrate concentrations (Fig 3A). Although at concentrations above 50 μM, there is a higher percentage of glucuronides, the remaining percentage of UCB is higher because of a slower rate of glucuronidation by enzymatic saturation. The kinetic profiles of bilirubin glucuronidation by RLM followed a Michaelis‒Menten model (Fig 3B). The data were transformed by the Lineweaver‒Burk equation (r^2^ = 0.9942), and the kinetic parameters calculated for km and Vmax were 41.42 ± 10.41 and 0.4211 ± 0.042 nmol/min/mg of protein, respectively.

A comparison of the results of in vitro glucuronide synthesis with those obtained in hyperbilirubinemic patients (S1 Fig) showed that the in vitro assays produced significantly fewer glucuronides than did the patients. In addition, at the end of the conjugation reaction, a significant amount of UCB is retained, which could interfere with the calibration curve and subsequent quantification. Consequently, the extraction and purification of BMG from patient samples were performed following the instructions outlined in the “bilirubin conjugates obtained from patient samples” section to develop and validate the analytical method.

### Method development

During the optimization of chromatographic conditions, several mobile phases prepared with different solutions (e.g., 0.1% or 1% formic acid in water, 2 mM, or 5 mM ammonium acetate, ACN, or MeOH) were tested individually or mixed in different proportions. In optimizing ionization (tuning), the best results were achieved with 5 mM ammonium acetate (pH 6) combined with ACN.

Dissolution tests shown that pure DMSO was the best solvent for the bilirubin standard, whereas a mixture of 20% DMSO and 80% MeOH plus ascorbic acid (100 mM) was optimal for the extraction of serum metabolites.

**Fig 4:**
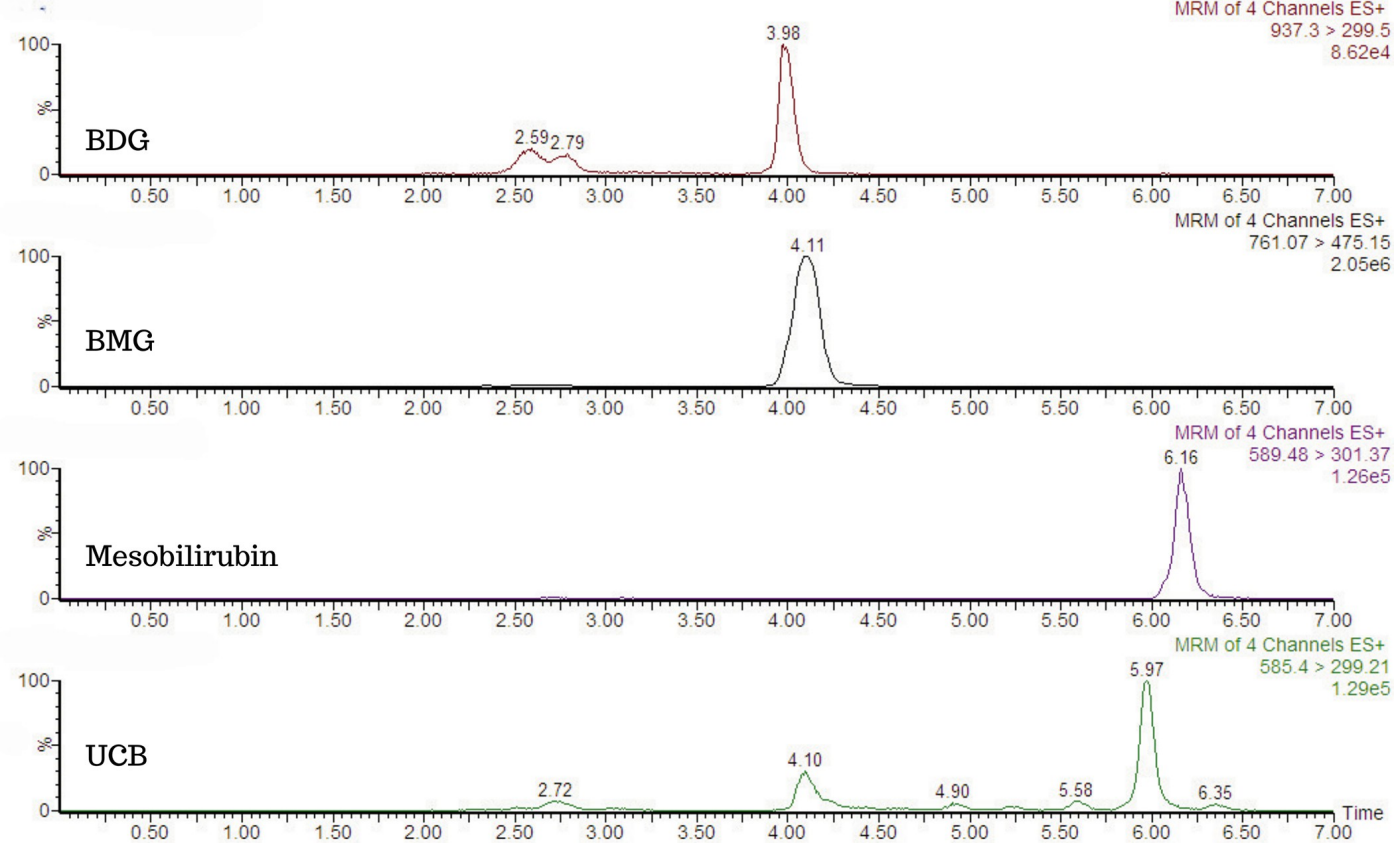
Bilirubin molecular species. m/z^1+^ specified for each species. Bilirubin unconjugated (UCB) was detected at 5.93 min (585.4 > 299.2), bilirubin monoglucuronide (BMG) at 4.11 min (761.3 > 475.3), bilirubin diglucuronide (BDG) at 3.98 min (937.3 > 299.5) and mesobilirubin as an IS at 6.16 min (589.4 > 301.3).

The final method allowed the detection of molecular species of bilirubin, with m/z^1+^, for UCB (585.4 > 299.2), BMG (761.3 > 475.3), BDG (937.3 > 299.5) and mesobilirubin as internal standards (589.46 > 301.37) and retention times of 5.97 min, 4.11 min, 3.98 min and 6.16 min, respectively (Fig 4).

### Validation parameters

#### Selectivity and carryover

No interference was observed for propofol, dexmedetomidine, ceftriaxone or prednisone for any of the analytes of interest. No carryover was observed for UCB, I.S. or any of the conjugates (S2 Fig).

#### Matrix effect

For this assay, quality control concentrations of UCB and BMG were used; the CV values of UCB were 1.13%, 1.04% and 1.43%, whereas for BMG, the CV values were 4.76%, 5.89% and 5.95%, respectively. Therefore, no matrix effect for either compound was observed.

#### Linearity

After three days of validation, the average of the curves analyzed revealed that the range of 10–60 μM/mL UCB was linear (r^2^ = 0.9966). For BMG, two ranges were tested: 3.84–122 μM/mL and 0.89–28.60 μM/mL (Table 2). Both showed linearity (r^2^ = 0.9989 and r^2^ = 0.9995, respectively).

**Table 2:** Linearity of the LC‒MS/MS method for the simultaneous quantification of UCB and BMG in serum.

**UCB concentration (μM)**						
	**10 μM**	**20 μM**	**30 μM**	**40 μM**	**50 μM**	**60 μM**
1	9.83	20.52	30.76	40.62	48.87	58.28
2	9.64	21.39	30.47	41.11	47.91	56.72
3	9.62	21.26	31.46	40.04	47.96	57.67
Mean (μM)	9.70	21.06	30.90	40.59	48.25	57.56
SD	0.12	0.47	0.51	0.54	0.54	0.79
CV (%)	1.20	2.23	1.65	1.32	1.12	1.37
Deviation %	**3.03**	-5.28	-2.99	-1.47	3.51	4.07
Equation	y = 9.4447x + 1.6176, r^2^ = 0.9966					
**BMG High range curve (μM)**						
	**3.84 μM**	**7.62 μM**	**15.25 μM**	**30.50 μM**	**61.01 μM**	**122.02 μM**
1	3.88	7.76	15.53	31.07	62.14	124.28
2	4.03	8.08	16.13	42.46	62.17	122.96
3	3.95	7.91	15.81	31.62	63.24	126.49
Mean (μM)	3.95	7.92	15.82	35.05	62.52	124.58
SD	0.08	0.16	0.30	6.42	0.63	1.78
CV (%)	1.90	2.02	1.90	18.33	1.00	1.43
Deviation %	-2.95	-3.89	-3.76	-14.92	-2.47	-2.10
Equation	y = 0.9845x + 0.9373, r^2^ = 0.9989					
**BMG Low range curve (μM)**						
	**0.89 μM**	**1.78 μM**	**3.57 μM**	**7.15 μM**	**14.30 μM**	**28.60 μM**
1	0.93	1.86	3.72	7.44	14.89	29.78
2	0.91	1.92	3.93	7.83	13.69	24.63
3	0.85	1.6	3.2	6.4	12.81	25.63
Mean (μM)	0.90	1.79	3.62	7.22	13.80	26.68
SD	0.04	0.17	0.38	0.74	1.04	2.73
CV (%)	4.64	9.49	10.39	10.23	7.57	10.24
Deviation %	-0.47	-0.75	-1.31	-1.02	3.52	6.71
Equation	y = 0.9490x + 0.2825, r^2^ = 0.9995					

SD: standard deviation; CV: coefficient of variation = [(standard derivation/mean)*100]; deviation [(theoretical concentration minus calculated concentration/theoretical concentration)*100].

#### Accuracy and precision

The accuracy and precision results are shown in Table 3, and both the UCB and BMG met the acceptance criteria.

**Table 3:** Results of the validation of the accuracy and precision of UCB and BMG in serum.

	**Intraday**			**Interday**			
**UCB**							
	**Mean ± SD**	**CV %**	**Deviation %**	**Mean ± SD**	**CV %**	**Deviation %**	**Recovery %**
**LLQ (10 μM)**	9.87 ± 0.43	4.35	1.27	9.79 ± 0.24	2.45	2.08	97.91
**LQC (15 μM)**	15.17 ± 1.13	7.50	-1.15	14.19 ± 1.48	10.49	5.37	94.62
**MQC (35 μM)**	34.70 ± 2.10	6.06	0.84	34.44 ± 0.95	2.76	1.59	98.40
**HQC (45 μM)**	44.55 ± 2.77	6.21	0.99	43.43 ± 1.47	3.39	3.47	96.52
**BMG (High range)**							
**LLQ (3.84 μM)**	3.84 ± 0.22	5.74	-0.03	3.95 ± 0.07	1.89	-2.85	102.85
**LQC (5.71 μM)**	6.21 ± 0.40	6.44	-5.00	6.02 ± 0.17	2.94	-5.48	105.48
**MQC (22.87 μM)**	22.08 ± 0.67	3.04	3.43	21.63 ± 0.32	1.50	5.39	94.61
**HQC (91.51 μM)**	91.35 ± 2.70	2.96	0.19	90.33 ± 1.85	2.05	1.28	98.71
**BMG (Low range)**							
**LLQ (0.89 μM)**	0.90 ± 0.02	1.97	-1.33	0.90 ± 0.03	2.81	-1.59	101.59
**LQC (1.39 μM)**	1.26 ± 0.10	8.06	8.80	1.19 ± 0.07	6.04	13.77	86.22
**MQC (5.58 μM)**	5.96 ± 0.31	5.28	-6.85	6.06 ± 0.21	3.49	-8.62	108.62
**HQC (21.5 μM)**	22.25 ± 0.45	2.03	-3.50	23.31 ± 0.52	2.33	-3.80	103.80

The intraday and interday assays. The standard deviation (SD), coefficient of variation (CV). Lower limit of quantification (LLQ), low-quality (LQC), medium-quality (MQC) and high-quality (HQC) controls concentrations were used for accuracy and precision tests.

#### Stability

The UCB solution remained stable after 4 hours in an autosampler at 21°C with light protection for 7 days at -80°C in the dark but was not stable when exposed to room temperature (25°C) or light or after three freeze‒thaw cycles (Table 4). The BMG solution was stable for 2 months when stored at -80°C and for 24 h in an autosampler at 21°C but was not stable at room temperature (25°C) when exposed to white light or after two freeze‒thaw cycles (Table 4).

**Table 4:** Storage conditions for UCB and BMG.

Storage condition	LQC (15 μM)	CV %	Deviation %	HQC (45 μM)	CV %	Deviation %
**UCB**						
**Freeze (7d)**	13.95±0.87	6.27	6.97	41.70±3.47	8.33	7.32
**Room (25°C)/2 h**	12.12±0.46	3.79	19.14	36.87±1.28	3.47	18.06
**Autosampler (21°C)/4 h**	12.71±0.89	7.02	15.25	36.53±4.91	13.44	18.81
**Freeze‒thaw (3 cycles)**	9.26±0.38	4.18	38.22	37.80±4.03	10.66	15.99
**BMG**						
**Storage Condition**	**LQC (5.71 μM)**	**CV %**	**Deviation %**	**HQC (91.51 μM)**	**CV %**	**Deviation %**
**Freeze (60d)**	5.20±0.05	1.03	8.90	82.55±1.37	1.66	9.79
**Room (25°C)/2 h**	4.55±0.26	5.89	20.25	76.63±1.63	2.13	16.25
**Autosampler (21°C)/24 h**	5.10±0.41	8.06	10.58	81.55±1.06	1.30	10.87
**Freeze‒thaw (2 cycles)**	5.26±0.36	6.95	7.71	88.71±1.10	1.25	3.05

The samples were frozen at -80°C, at room temperature (25°C exposed to white light), under an autosampler (21°C, light protection), and subjected to freeze‒thaw cycles (stored at -80°C for 24 h and thawed at 25°C). LQC and HQC were evaluated under all storage conditions.

As previously stated, BDG quantification was not performed due to difficulties in the import process of this standard; however, the molecule was successfully identified (transition 937.3 > 299.5) to allow the quantification of BDG in patient samples by the correction factor reported by Putluru et al., 2016 [31]. Notably, all the molecular species were estimated with this correction factor for comparison with the concentrations determined with our method.

### Method application

**Fig 5:**
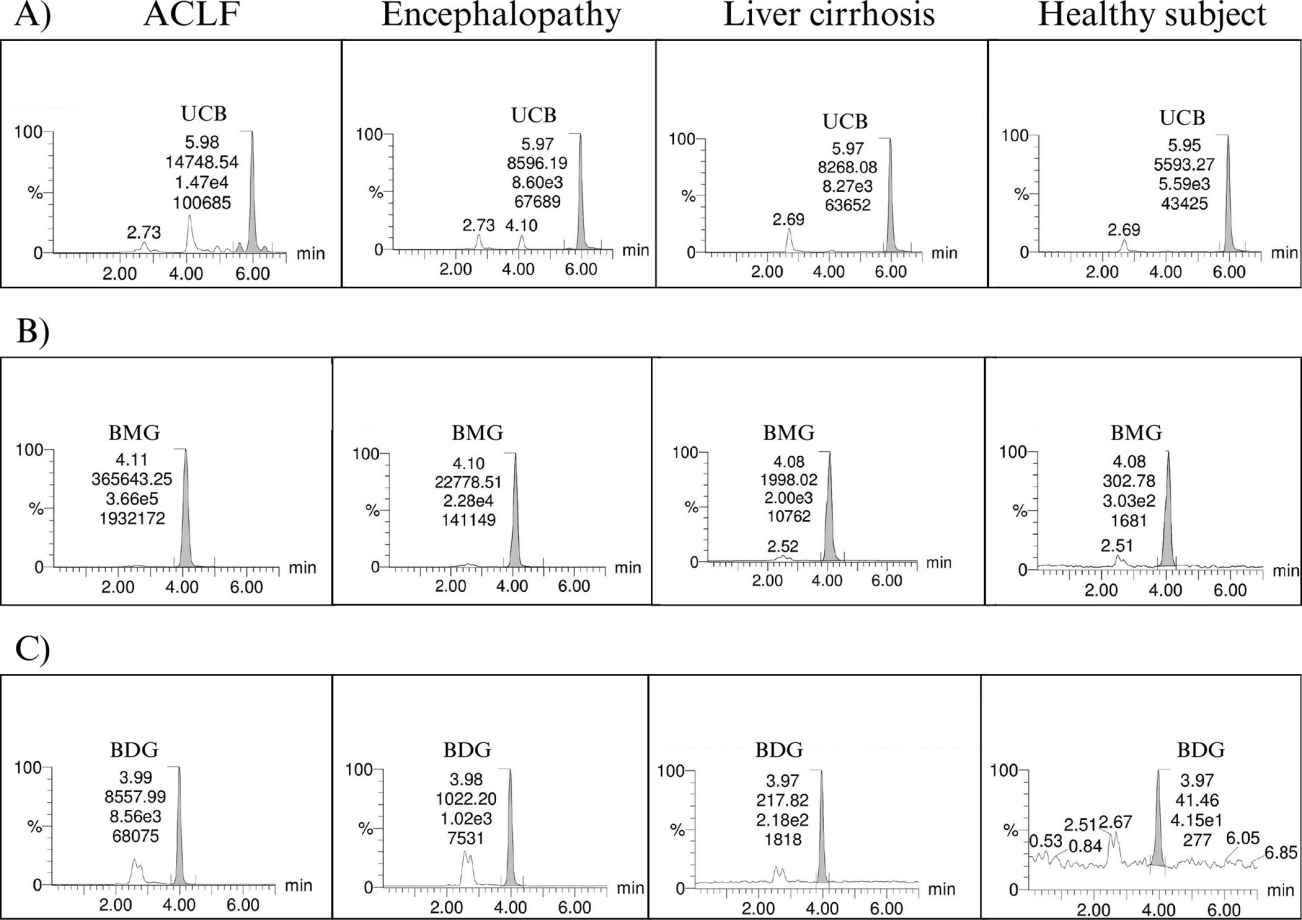
m/z scan showing the molecular species of bilirubin in positive mode for each type of patient with liver disease: Acute-on-chronic liver failure, hepatic encephalopathy, liver cirrhosis, and healthy subjects. A) Integrated m/z scan of UCB from one patient for each group. B) Integrated m/z scan from BMG for each group. C) Integrated m/z scan from the BDG for each group.

Patients with liver diseases associated with high bilirubin concentrations, including acute-chronic liver failure, hepatic encephalopathy and compensated cirrhosis, and a control group of healthy subjects with normal bilirubin levels were recruited. In the patients’ serum, three molecular species of bilirubin were identified by their m/z, as shown in the representative spectrograms in Fig 5. Table 5 presents the concentrations of UCB, BMG, and BDG, alongside the experimental results and those calculated with a correction factor [31]. Owing to the unavailability of the BDG standard and the impracticality of purifying it from samples, BDG was quantified by the UCB calibration curve. Patients with more advanced liver damage had higher serum BDG concentrations, with the acute-on-chronic liver failure group having the highest levels of all bilirubin species.

**Table 5:** Serum concentrations of patients with acute-on-chronic liver failure, hepatic encephalopathy, and liver cirrhosis and healthy subjects were obtained experimentally and with a correction factor.

Group	Bilirubin molecular specie	Experimental result (μmol/L)	Correction factor (μmol/L)	p value	Critical value
Healthy subjects	BDG	0.031 (0.022–0.048)	0.008 (0.005–0.032)	0.017	0.017
	BMG	0.564 (0.342–0.765)	0.095 (0.064–0.128)	0.005	0.004
	UCB	7.158 (4.902–11.932)	0.879 (0.644–1.418)	0.005	0.008
Liver cirrhosis Compensated	BDG	0.052 (0.036–0.104)	0.028 (0.018–0.396)	0.735	0.050
	BMG	1.301 (0.370–4.279)	0.190 (0.113–0.438)	0.018	0.021
	UCB	11.203 (6.852–18.083)	9.910 (6.830–21.780)	0.441	0.038
Hepatic encephalopathy	BDG	0.028 (0.018–0.396)	0.888 (0.070–1.899)	0.066	0.025
	BMG	0.372 (0.190–1.113)	2.129 (0.328–4.879)	0.110	0.033
	UCB	8.106 (4.220–19.263)	13.960 (12.110–30.255)	0.086	0.029
Acute-on-chronic liver failure	BDG	1.038 (0.565–3.635)	0.991 (0.503–8.351)	0.646	0.042
	BMG	17.432 (9.687–76.385)	1.325 (0.498–14.190)	0.005	0.013
	UCB	23.734 (7.839–53.004)	14.797 (6.671–38.817)	0.674	0.046

Unconjugated bilirubin (UCB), bilirubin monoglucuronide (BMG) and bilirubin diglucuronide (BDG). p value was determined by the Wilcoxon signed-rank test. Critical value by Benjamini‒Hochberg.

## Discussion

The primary objective of this study was to develop a sensitive and specific method for the identification and quantification of the molecular species of bilirubin, BMG, BDG, and UCB by LC‒MS/MS. Our findings yield several important insights. Notably, patients with liver failure present elevated bilirubin levels (hyperbilirubinemia). Although evaluating hyperbilirubinemia can be complex, expensive, and sometimes invasive, as it can include, in addition to routine laboratory tests, imaging examinations, histological examinations, and/or gene mutation [3], LC‒MS/MS offers a more accurate and specific measurement of bilirubin species than traditional clinical methods do. However, the precise quantification of glucuronidated bilirubin species is frequently limited by the difficulty of obtaining pure standards. Methods such as those described by Blanckaert et al. [38] use alkaline methanolysis to efficiently convert conjugated bilirubin to more easily separable methyl esters, allowing highly specific quantification of mono- and di-conjugates in biological samples. While accurate, this method can be complex and lacks the ability to directly identify the conjugating sugars. Consequently, methods have been developed for the in vivo or in vitro synthesis of these species by purified enzymes or HLMs or RLMs to produce bilirubin conjugates. In addition, previous studies have shown that enzyme systems such as UDP-glucuronosyltransferase are critical for the conversion of bilirubin to its conjugated forms, as shown in microsomal preparations from rat liver [39]. In our study, the optimization of the conjugation conditions described in the Methods section was sufficient to obtain bilirubin monoglucuronide, similar to the efficiency of conjugate production observed in Blanckaert’s in vitro models.

To this end, various parameters, including the UCB concentration, microsomal protein concentration, UGT1A1 concentration, incubation time, and UDPGA (glucuronic acid group donor) concentration, were explored [32]. Enzyme induction with omeprazole was successful in increasing UGT1A1 expression. However, upon protein quantification, the concentrations of the HLMs were lower than those of the RLMs. Despite this difference in protein concentration, bilirubin conjugation was performed using both types of microsomes. This procedure allowed a comparison of the glucuronidation efficiency between RLMs and HLMs, and the results showed that the RLM system exhibited superior performance compared with that of HLM, as the BMG peak was superior to that of RLM. Notably, the concentration of BMG was greater in RLM than in HLM, suggesting that rat liver microsomes exhibit superior glucuronidation efficiency for this substrate, as they exhibit differences in enzyme activity, substrate affinity, and metabolic rates. These differences may be attributed to species-specific variations in UGT1A1 activity that affect the metabolic processing of bilirubin. These differences indicate the limitations of using the RLM as a model for human liver metabolism, as the enzymatic behavior and metabolic profiles in rats do not fully replicate those in humans. In contrast, the chromatographic peak corresponding to BDG was not observed when it was analyzed by LC‒MS/MS, indicating that this metabolite is either not formed or that its concentration is below the limit of detection, thus preventing its identification or quantification. As demonstrated by Blanckaert [39], the absolute amount of BDG or BMG synthesized changed little when the bilirubin concentration was increased. Consequently, the formation of BMGs by microsomal preparations was greater than that of BDGs when high bilirubin substrate concentrations were used. Although they reported that almost equal proportions of BMG and BDG were synthesized by microsomes when the substrate concentration was lower, we could not demonstrate that BDG was formed. Therefore, the use of RLM and HLM is not a viable approach for obtaining the metabolite in question. The limitations of these models can be attributed to differences in the expression of UGT1A1 between different subjects, different tissue preparations, and cell lines, which can vary over time. Additionally, a microsome preparation is only capable of yielding moderate percentages of the total enzymes present in the tissue [24, 33]. In contrast, BDG was observed in some patient samples by the transition described by Gong et al. [40], thereby corroborating the identification of the peak in our samples as belonging to BDG. On the basis of the above observations, our method is suitable for the identification and quantification of BDG.

Although bilirubin metabolites have been quantified by conventional HPLC [30, 38, 41], mass spectrometry, and LC/MS [22, 24, 25], these studies used the HLM system and the recombinant enzyme UGT1A1 [24, 31, 40] to obtain conjugates, which proved to be an inefficient system. Adachi [42] noted that the stability of bilirubin glucuronides, especially under HPLC conditions, is a critical consideration. Their work demonstrated that without proper stabilization, such as the use of reducing agents such as ascorbic acid, bilirubin monoglucuronide (BMG) can degrade, complicating the quantification process. Therefore, we tested an alternative method to obtain BMG from patient samples, which yielded greater amounts of the compound than microsomes did. In addition, separating BMG by HPLC allows us to obtain a pure compound, ensuring that the calibration curves and the quantification of serum levels accurately reflect the true concentrations present in the samples.

On the other hand, several studies have indirectly quantified the levels of the conjugates by assuming that the conjugates have molar absorptivities identical to those of UCB [26, 43]. However, accurate identification and quantification of bilirubin and its glucuronides require rigorous chromatographic conditions, including selectivity to differentiate analytes, sensitivity to detect minimal concentrations, and efficient extraction from biological samples. These critical factors, such as solubility, polarity, and ionization degree, are often overlooked in other methods described in the literature [27, 28]. Most previous studies have focused primarily on analyzing conjugates in bile and serum from healthy subjects, with many of these studies dating back to the 1980s and 1990s.

However, our study represents a significant advance because we directly measured serum bilirubin conjugates (BMG and BDG) instead of relying on UCB calibration curves. This direct quantification was made possible by the use of mass spectrometry, which provides superior sensitivity at low concentrations [31, 33]. In addition, we optimized analyte solubility using a mixture of MeOH and DMSO, which enhanced recovery by preventing bilirubin from binding to albumin and ensuring a protein-free extraction solution. Incorporation of ascorbic acid further stabilized the conjugates by preventing oxidation. Compared to other methods, our approach stands out for its simplicity and efficiency. While Wang et al. [24] used MeOH-ACN mixtures and temperature-controlled centrifugation, which provided excellent control over protein precipitation, their process required 30 minutes of centrifugation. In addition, their focus on enzymatic activity through microsomal incubation is more appropriate for metabolic studies rather than simple analyte quantification. The method of Martelanc et al. [44] used ultracentrifugation for optimal sample purity, but this adds complexity and may result in analyte loss, especially for bilirubin, due to the longer process and dilution steps. While Zelenka et al. [45] used a complex mixture of chloroform, methanol, and hexane along with pH adjustments to ensure high recovery, our use of a simple mixture of methanol and DMSO provides a similar benefit by increasing solubility and preventing bilirubin from binding to albumin, but with fewer steps. Additionally, unlike Itoh et al [43], whose extraction focuses primarily on separating bilirubin photoisomers using ACN and DMSO, our method is designed to achieve a protein-free extraction, ideal for stabilizing bilirubin conjugates. Thus, our method balances efficiency, speed, and high recovery with minimal sample loss while maintaining sufficient sensitivity for accurate analyses, making it well suited for routine analysis of bilirubin and its conjugates in clinical and experimental settings. Importantly, our method highlights the critical role of temperature control, as maintaining samples at 21°C prior to analysis avoids irreproducibility caused by increased viscosity when temperatures fall below this threshold.

After the levels of the samples from both healthy subjects and patients were analyzed, we compared the levels obtained with our quantification method and the mathematical method using the correction factor. For BDG, significant differences were found only in the healthy group when the concentration was estimated. This may be due to the use of the correction factor and the mathematical estimation using the UCB calibration curve, which requires the use of a specific curve for this purpose. In contrast, BMG levels were significantly greater in patients with acute-chronic liver failure, which was accurately detected by our method. Although the concentration of UCB was measured by a calibration curve with a reference standard, the observed differences in levels were statistically significant, which may be attributed to the use of two different methods, LC‒MS/MS and UV‒HPLC, as well as the relatively small sample size of 10 patients. A comparison of the levels obtained with the correction factor and those obtained with the curve calibration shown statistically significant differences (p < 0.05) according to the Wilcoxon signed-rank test. The correction factor underestimated the levels in most groups, including healthy subjects, patients with cirrhosis, and patients with ACLF. However, in patients with hepatic encephalopathy, the estimated levels were higher than those measured experimentally using the calibration curve. The results obtained after applying the BH procedure highlight the variability that correction factors can introduce into the quantification of bilirubin species after finding 4 significant results: BMG and UCB in healthy subjects, BMG in compensated cirrhosis patients and BMG in acute-on-chronic liver failure patients. This suggests that BMG is particularly sensitive to the methodological differences introduced by correction factors. This finding supports the premise that direct quantification by LC‒MS/MS offers distinct advantages over correction factor-based approaches. By directly measuring bilirubin molecular species, we eliminate the potential for correction-induced variability, leading to more accurate and reproducible results.

The integration of LC‒MS/MS into clinical practice holds great promise for improving the diagnosis and management of liver disease by providing accurate quantification of bilirubin molecular species and other biomarkers, such as bile acids [46], oxylipins [47] and proteins such as B2M, IGFBP3, IGFALS [48] and ApoA1 [49]. LC‒MS/MS has already played a transformative role in the diagnosis of liver disease by enabling the simultaneous detection of multiple biomarkers in a single analysis, providing a comprehensive understanding of complex liver disease pathways, including hepatitis-induced injury and alcoholic liver disease. This method offers advantages over traditional techniques such as ELISA, with faster and more accurate biomarker detection in diseases such as hepatitis, cirrhosis and hepatocellular carcinoma [49]. However, to realize its full potential, larger clinical studies are needed to validate its use in diverse populations, establish clinically relevant reference ranges, and ensure standardization between laboratories. The development of uniform protocols for sample preparation, extraction, and analysis is critical for achieving consistent results. With further validation, LC‒MS/MS could become a reliable tool in clinical practice, offering more accurate monitoring of liver function and disease progression, ultimately enabling more tailored treatment strategies.

This is the first study to directly identify and quantify the molecular species of bilirubin in various liver diseases by LC‒MS/MS. Previous studies have typically estimated conjugate levels on the basis of the molar extinction coefficient of UCB, assuming that it is the same for bilirubin glucuronides [22, 24]. Other researchers have used the diazo method to quantify total bilirubin and then multiplied the peak areas of each molecular species of bilirubin [26]. Although mass spectrometry has been used in several studies to identify BMG, BDG, and UCB, most have focused on identification rather than quantification, often evaluating the m/z signal ratio of BDG and BMG to infer the value of conjugated bilirubin [40].

By accurately measuring the individual molecular species of bilirubin, including BDG, BMG, and UCB, this approach provides a more precise understanding of bilirubin metabolism and its disruption in liver disease. Such detailed quantification is particularly important in hyperbilirubinemia, where the levels of these metabolites can serve as biomarkers for the extent of liver damage, as observed in conditions such as acute‒chronic liver failure and cirrhosis. Direct measurement of these species eliminates the uncertainties associated with indirect methods, enabling clinicians to better assess disease severity and progression.

While our methodology is based on the LC-MS/MS technique initially described by Putluru et al., important modifications have been made. The calculated correction factor may vary between laboratories or even between different replicates of the same experiment, and factors that could influence the values when using the correction factor may be associated with the type of sample preparation, the extraction procedure used, the stability of the sample, as well as the analytical conditions. These variations may lead to inconsistencies in the corrected BDG levels, which could disproportionately reduce or increase BDG levels, leading to possible misinterpretation of their relevance in pathological processes. To minimize possible errors, it is essential to use calibrators with known concentrations, using the DBG standard, which is marketed as bilirubin dimethyl ester. Although we faced difficulties in importing it, with our method we were able to identify and extract it from patient samples, as shown in the spectrograms. Therefore, anyone interested in quantifying DBG and who can obtain this standard will be able to apply the methodology presented, using the calibration range that we propose based on the results of this study.

In contrast, our method provides a significant improvement by directly identifying and quantifying molecular bilirubin species, eliminating the need for a correction factor. This direct quantification minimizes the risk of variability and ensures more reliable and consistent results across studies and settings. Furthermore, while Putluru et al. used a combination of LC-MS/MS and UV-HPLC methods, we achieved the same precision and accuracy using LC-MS/MS alone. This single-method approach simplifies the process, reduces potential methodological inconsistencies, and optimizes sample analysis. It is important to note that using calibrators with known concentrations, the results tend to be more accurate than those obtained by calculations. This is because calculations often involve two different methods with different principles: LC-MS/MS is based on the mass-to-charge ratio, while UV spectroscopy measures light absorption, which can introduce biases.

In addition, because no previous studies have reported the levels of these bilirubin conjugates in hyperbilirubinemic biological samples, we are unable to directly compare our results with those of other clinical studies. This highlights the need for further research with larger sample sizes to better understand the role of these metabolites in liver disease. By comparing our LC-MS/MS-based method with the approach of Putluru et al. using their correction factor as a reference point for standardization, we can assess the robustness of our method and its potential for broader clinical applications. In this comparison, the values obtained by interpolation of the problem samples on the calibration curve and those derived using the correction factor were shown. The interpolated values fall within the validated range of linearity, while the values obtained using the correction factor are higher, indicating an overestimation of concentrations. This demonstrates that the values calculated using the correction factor are not adequate for accurate quantification. Our method not only enhances reproducibility but also offers a more robust framework for future clinical applications. This comparison also enables us to refine the quantification ranges for bilirubin species, as suggested by our findings. Specifically, we propose that UCB should be quantified between 5 and 50 μM, BMG in two ranges from 0.5 to 25 μM and 25 to 50 μM, and BDG between 0.02 and 5 μM. These proposed ranges address a critical gap in the literature and offer a more direct measurement that provides greater clarity in assessing bilirubin metabolism and its role in liver diseases, making it a more effective and reliable tool for both research and diagnostic purposes.

## Conclusion

This study is the initial effort to directly assess the levels of molecular species of bilirubin in human blood serum through liquid chromatography‒mass spectrometry. The use of standards extracted from hyperbilirubinemic serum is proposed to circumvent the need for calculations and correction factors. Despite our use of in vitro glucuronidation, we found that the BMG extracted from serum exhibited superior performance in the quantification process. Considering the necessity for timely and effective identification and quantification in critical patients, such as those with cirrhosis who develop ACLF, the LC‒MS method allows for the precise determination of each molecular species of bilirubin. This could improve the diagnosis and prognosis of patients by identifying which species is more effective, thereby facilitating the initiation of specific and target-specific treatments.

## Supporting information

S1 Fig Molecular species of bilirubin in microsomes and human serum. Panel A) Chromatogram showing the peaks resulting from the incubation of microsomes with unconjugated bilirubin (UCB) for 20 minutes to obtain bilirubin monoglucuronide (BDG) and bilirubin diglucuronide (BDG), whereas Panel B) shows the peaks of bilirubin conjugates purified from patient samples. The transitions for each molecular species of bilirubin were as follows: BDG m/z 937.33 > 299.5, BMG 761.30 > 274.30, and UCB 585.27 > 299.21.
(TIFF)

S2 Fig Carryover of UCB and BMG. Panel A shows the carryover of UCB with a previously injected blank sample chromatogram, a UCB high concentration level and a blank sample injected after the high concentration. Panel B shows the carryover of BMG with a previously injected blank sample chromatogram, a BMG high concentration level and a later injected blank sample.
(TIF)

S1 Dataset (XLSX)
