# Supplementary figures and images for: Identification and quantification of the molecular species of bilirubin BDG, BMG and UCB by LC‒MS/MS in hyperbilirubinemic human serum
Source: PLoS One. 2024 Nov 19;19(11):e0313044. doi: 10.1371/journal.pone.0313044 (PMC11575834; doi:10.1371/journal.pone.0313044)

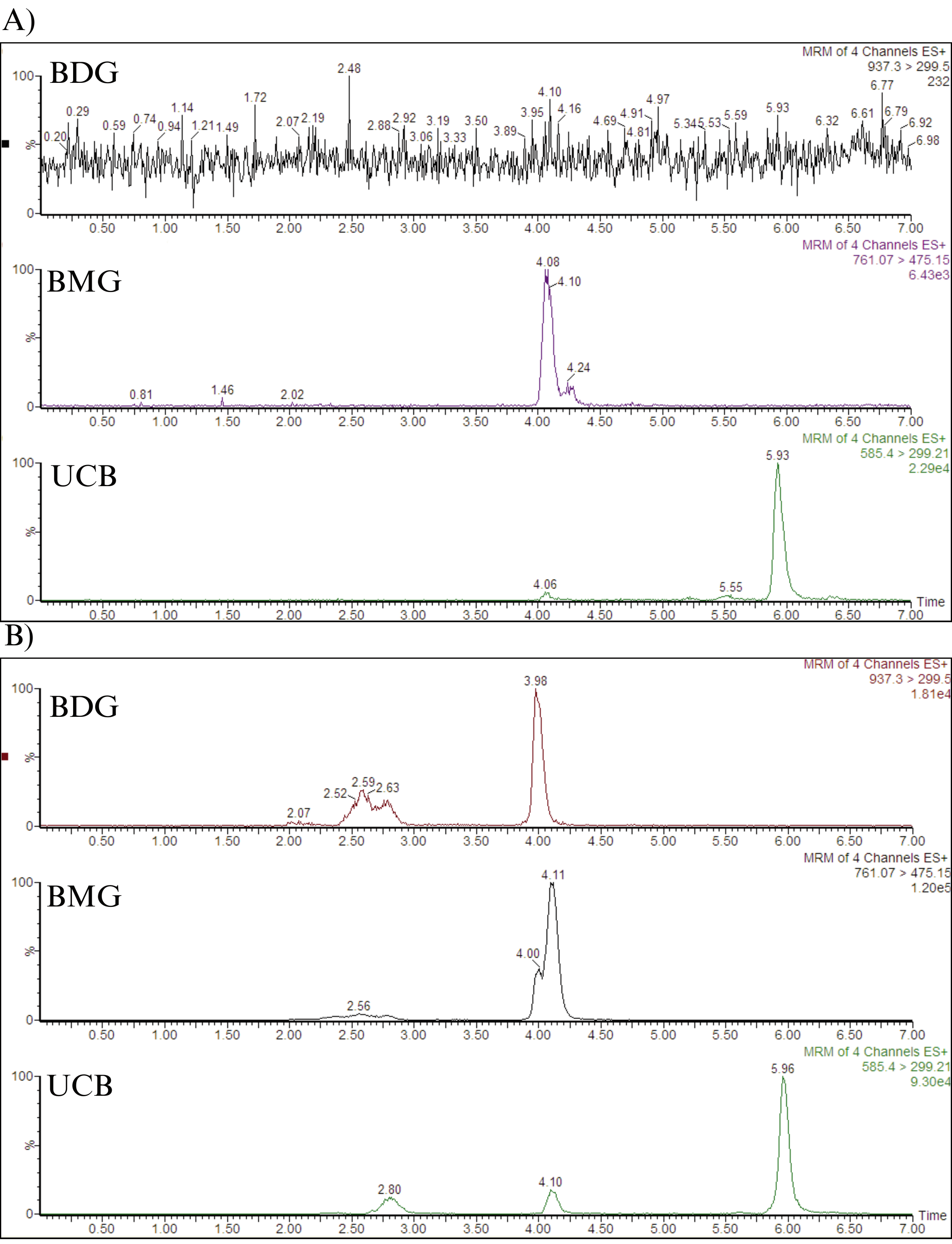

Supplement: S1 Fig — Panel A) Chromatogram showing the peaks resulting from the incubation of microsomes with unconjugated bilirubin (UCB) for 20 minutes to obtain bilirubin monoglucuronide (BDG) and bilirubin diglucuronide (BDG), whereas Panel B) shows the peaks of bilirubin conjugates purified from patient samples. The transitions for each molecular species of bilirubin were as follows: BDG m/z 937.33 > 299.5, BMG 761.30 > 274.30, and UCB 585.27 > 299.21. (TIFF) [file pone.0313044.s001.tiff]

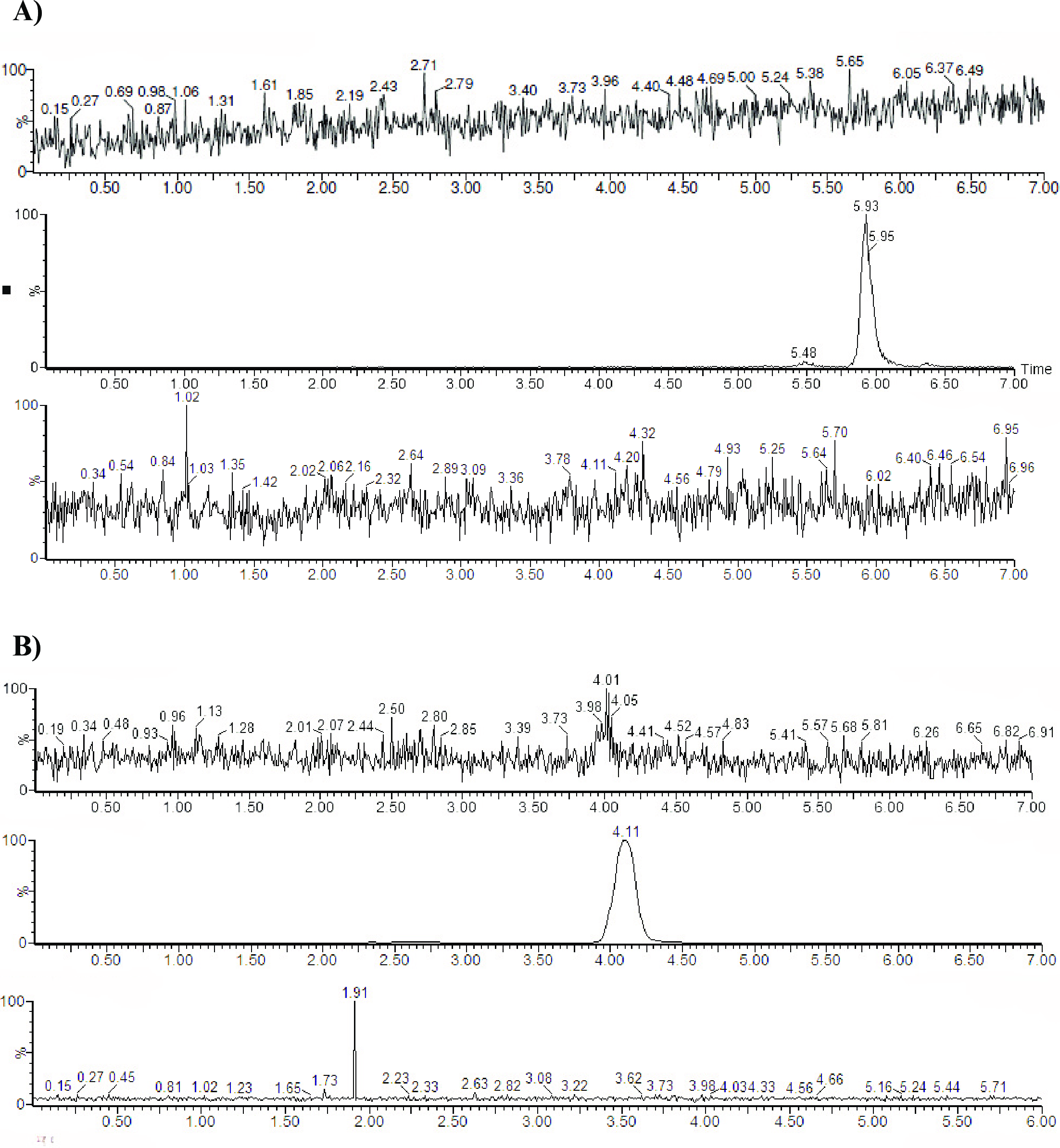

Supplement: S2 Fig — Panel A shows the carryover of UCB with a previously injected blank sample chromatogram, a UCB high concentration level and a blank sample injected after the high concentration. Panel B shows the carryover of BMG with a previously injected blank sample chromatogram, a BMG high concentration level and a later injected blank sample. (TIF) [file pone.0313044.s002.tif]
